# Alterations of Golgi Structural Proteins and Glycosylation Defects in Cancer

**DOI:** 10.3389/fcell.2021.665289

**Published:** 2021-05-12

**Authors:** Xiaoyan Zhang

**Affiliations:** ^1^College of Biomedicine and Health, Huazhong Agricultural University, Wuhan, China; ^2^College of Life Science and Technology, Huazhong Agricultural University, Wuhan, China

**Keywords:** glycosylation, cancer, Golgi fragmentation, Golgi matrix, GM130, GRASP55, COG complex

## Abstract

As the central hub in the secretory and endocytic pathways, the Golgi apparatus continually receives the flow of cargos and serves as a major processing station in the cell. Due to its dynamic nature, a sophisticated and constantly remodeling mechanism needs to be set up to maintain the Golgi architecture and function in the non-stop trafficking of proteins and lipids. Abundant evidence has been accumulated that a well-organized Golgi structure is required for its proper functions, especially protein glycosylation. Remarkably, altered glycosylation has been a hallmark of most cancer cells. To understand the causes of Golgi defects in cancer, efforts have been made to characterize Golgi structural proteins under physiological and pathological conditions. This review summarizes the current knowledge of crucial Golgi structural proteins and their connections with tumor progression. We foresee that understanding the Golgi structural and functional defects may help solve the puzzle of whether glycosylation defect is a cause or effect of oncogenesis.

## Introduction

Cancer ranks as one of the leading causes of death globally. Governments, research institutes, and pharmaceutical firms spend billions of dollars into the fight against this terrible human disease. Even with the pleasing progress in the tumorigenesis theory, the powerful therapy is still limited due to several real difficulties. The majority of tumors are detected at an advanced stage, leading to unsuccessful targeted treatment. Remarkably, the existence of tumor heterogeneity and cancer stem cells is largely accounted for tumor recurrence and later drug resistance (Barkeer et al., 2018). Despite the significant improvement of traditional methods, such as chemotherapy, radiation therapy, surgery, and the advent of the targeted therapy, only a tiny percentage of patients benefit (Ayyar et al., 2016; Costa et al., 2020). Therefore, the identification of common and reliable targets and the development of related new-targeted therapies are crucial for cancer study and the drug industry (Costa et al., 2020).

The discovery of the difference between normal and tumor cells is the key for targeted therapy. Fortunately, altered glycosylation of cell surface proteins and lipids, and/or extracellular vesicles, are well accepted as a hallmark of cancer cells (Pinho and Reis, 2015; Martins et al., 2021). However, the complexity of the glycosylation process and the underlying regulatory mechanisms make it up-to-date a tough nut to crack for deep investigation. Glycosylation is the most complicated and diverse post-translational modification (PTM), with the attachment of multiple kinds of glycans (carbohydrates) to proteins and lipids. It requires coordinated actions of different glycosyltransferases, glycosidases, nucleotide sugar transporters, and appropriate substrates in an orchestrated manner (Moremen et al., 2012; Hennet and Cabalzar, 2015). Unlike other general types of PTMs, such as phosphorylation and ubiquitination, which occurs in the cytosol or nucleus, the majority of glycosylation processes, except *O*-GlcNAcylation, happen in the lumen of membranous organelles, the endoplasmic reticulum (ER) and the Golgi apparatus (or the Golgi for simplicity). The limited and narrow space of ER/Golgi lumen may promise the efficacy and accuracy of this complicated modification which function in various crucial cellular events, such as signaling, cell–cell communication, cell-extracellular matrix (ECM) interaction (Moremen et al., 2012). The fact that altered Golgi structure and protein glycosylation pattern have been widely observed in various cancer types prompts researchers to study the mechanism of how Golgi structure regulates protein glycosylation. In this review, we will summarize the recent findings of the correlation between changes of Golgi structural proteins and alterations of glycosylation in tumorigenesis and comment on the enigmatic connection between Golgi morphological alteration and tumor development, aiming to provide insight that may help develop novel cancer therapies by targeting Golgi structural and functional defects.

## Glycosylation Is a Common and Crucial Modification

Glycans present in all known living organisms and are as essential as nucleic acids, proteins, lipids, and metabolites (Varki, 2017). In mammals, monosaccharides are linked via a glycosidic bond to form branched or unbranched chains. Such complex glycan linkages are attached to various macromolecules to generate glycoproteins, glycolipids, GPI-anchored proteins, and proteoglycans, which most often cover the cell surface or are secreted as extracellular materials. Except for the single sugar modification *O*-GlcNAcylation, which refers to the addition of β-*N*-acetylglucosamine (GlcNAc) onto serine or threonine residues (Ser/Thr) of the targeted protein in the cytosol or nucleus (Bond and Hanover, 2015), oligosaccharides on proteins are primarily divided into two categories, *N*-linked glycans and *O*-linked glycans, both of which are commonly found on secretory proteins and the extracellular domains of integral membrane proteins.

About half of all human proteins are glycoproteins, and most of them are *N*-glycosylated (Apweiler et al., 1999). *N*-glycans are synthesized as a lipid-linked oligosaccharide (LLO) precursor. When a new protein is synthesized, the 14-sugar chain GlcNAc2Man9Gluc3 of the LLO is transferred by the oligosaccharyltransferase (OST) to the amide group of the asparagine residue in the Asn-X-Ser/Thr motif, where X is any amino acid except proline (Kelleher and Gilmore, 2006). Before the glycoprotein leaves the ER, all three glucose residues and one particular mannose residue are removed. The resulting *N*-glycans are referred to as the high-mannose subtype and further trimmed in the *cis-*Golgi. Subsequently, the decoration of GlcNAc on mannose yields sugar branches in the *medial-*Golgi. Attachment of galactose, sialic acid, and fucose in the *trans-*Golgi generates complex *N*-glycans (Stanley et al., 2009). One single protein could possess multiple sugar chains added to different amino acids. Notably, sugar chains could be processed diversely, resulting in hybrid *N*-glycans which harbor both high-mannose and complex characteristics (Ungar, 2009). Therefore, the precise *N*-glycan modifications are generated by the accurate removal and addition of sugars in the Golgi, depending on the sequential distribution of the glycosylation enzymes in different cisternae. The same holds true for the nucleotide sugar transporters, which need to be precisely present in the right cisternae (Hirschberg et al., 1998).

Unlike the ER origination of *N*-glycosylation, *O*-glycosylation is more diversified and predominantly processed in the Golgi. There are two main types of *O*-glycans in mammalian cells: the matrix glycosaminoglycan (GAG) chains on proteoglycans and the most common mucin-type glycans (Brockhausen et al., 2009). The repeating disaccharides of unbranched GAG chains are attached to the serine residues on the core proteins of proteoglycans through a common tetrasaccharide linker (xylose–Gal–Gal–glucuronic acid). The disaccharide units contain either *N*-acetylgalactosamine (GalNAc) or GlcNAc, and a uronic acid, which are extended in the earlier Golgi. The frequent sulfation modification on the disaccharide then occurs in the *trans*-Golgi (Stanley, 2011). Mucin-type *O*-glycosylation initiates with the attachment of a GalNAc onto the Ser/Thr residues in a glycoprotein to form GalNAcα1-Ser/Thr, which is also called the tumor-associated Tn antigen (Ju et al., 2014). A family of enzymes known as polypeptide GalNAc-transferases (ppGalNAcTs) catalyzes this reaction. The Tn antigen is usually a precursor and followed by the addition of galactose, GlcNAc, or GalNAc to form core *O*-glycan structures. The critical step is to transfer galactose by the enzyme termed T-synthase (Core 1 β3-galactosyltransferase, C1GalT1) to form the common core 1 *O*-glycan (or the T antigen). The T antigen can be further processed into core 2 by core 2 GlcNAc transferases (C2GnTs) to generate GlcNAcβ1–6(Galβ1–3) GalNAcα1-Ser/Thr. The core 1 and/or core 2 *O*-glycans are ubiquitously expressed in humans. Although *O*-glycan structures are generally shorter than *N*-glycans, core 1–4 structures could be further extended to generate diverse glycan chains, such as polyLacNAc, Lewis antigens, and different blood group antigens (Kudelka et al., 2015).

## Aberrant Glycosylation Is a Hallmark of Cancer

Aberrant glycosylation frequently occurs in cancer, playing pivotal roles in cancer progression and metastasis, cell–cell interaction, and epithelial-mesenchyme transition (EMT) (Taniguchi and Kizuka, 2015). Altered glycosylation associated with cancer usually includes the overexpression of Tn and T antigen, and their sialylated counterparts (Sialyl-Tn), sialylated Lewis blood group (SLe^*a*^ and SLe^*x*^), as well as complex branched *N*-glycans, including β1,6-GlcNAc branching, bisecting GlcNAc, and core fucose (Taniguchi and Kizuka, 2015; Schneider et al., 2017; Peixoto et al., 2019). These features are most often observed in advanced solid tumors and often correlate with poor survival, indicating that universal mechanisms need to be uncovered. Genetically, protein levels of the responsive glycosylation enzymes are frequently reported dysregulated. For instance, upregulated expression of sialyltransferases seems to be a major cause for SLe^*a/x*^ expression (Kudelka et al., 2015; Bhide and Colley, 2017). Besides, altered expression of the active T-synthase or Cosmc, an ER-localized molecular chaperone required for the stability and activity of T-synthase, can lead to the overexpression of the Tn antigen (Ju et al., 2014). Other than the genetic regulation, the localization changes of glycosylation enzymes could also cause adverse expression of Tn antigen. The activation of the proto-oncogene tyrosine kinase Src could stimulate the COPI trafficking machinery-dependent relocation of GalNAc-transferases (GALNTs) from Golgi to the ER, which leads to increased activity of GALNTs and enhanced cellular Tn level. Therefore, the GALA (GALNT Activation) pathway provided a mechanism of how the membrane trafficking modulates protein glycosylation (Gill et al., 2010; Bard and Chia, 2016). Remarkably, ER-localization of GALNTs in the GALA pathway induces *O*-Glycosylation of the matrix metalloprotease MMP14 and ER-resident Calnexin, thus drives MMP14 activation and Calnexin/ERp57 cell surface distribution, respectively, both of which promote ECM degradation and tumor development (Nguyen et al., 2017; Ros et al., 2020). Collectively, the GALA pathway shed light on a complete picture of how the signal of oncogene converts to Golgi glycosylation enzyme mis-location, and finally, a vicious circle of tumor development. However, therapeutical options by targeting the featured antigens on cancer cells are very limited due to the complexity of glycans on the cell surface. More information on the underlying mechanisms that regulate glycosylation is deeply needed for future targeting.

Glycan processing certainly has to be tightly supervised. Different from proteins and nucleic acids, glycan structures are not template encoded. Intriguingly, glycosylation enzymes tend to form homomers, or a variety of functionally relevant acting glycosyltransferases form heteromers for sequential modification, providing a regulation mechanism from the enzyme part (Kellokumpu et al., 2016; Khoder-Agha et al., 2019). Remarkably, as the major manufacturing and supply chain, the Golgi has evolved a specific structure to promise proper protein glycosylation.

## Changes of Golgi Structural Proteins in Cancer Cells

The Golgi functions as the center in the secretory pathway and receives the uttermost cargos from the ER, modifies and sorts them inside or outside the cell. The cargos, including proteins and lipids, are subjected to extensive Golgi modifications, such as glycosylation, sulfation, phosphorylation, and proteolysis. The Golgi has evolved a particular stacked and ribbon-like structure in mammalian cells to perform its crucial functions. Each polarized stack is usually surrounded with transport vesicles and defined with three separate modules: the *cis*-Golgi network (CGN); the stacked *cis-, medial-*, and *trans-*Golgi cisternae; and the *trans*-Golgi network (TGN) (Klumperman, 2011; Day et al., 2013). Each cisterna was specialized with distinct enzymes for sequential modification of appropriate substrates.

**FIGURE 1:**
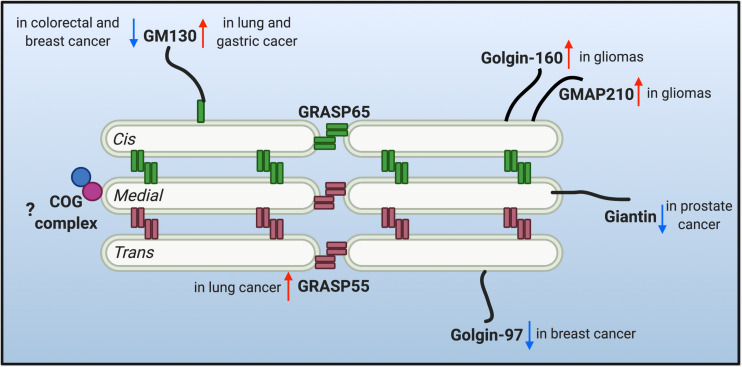
Protein level alteration of the reported Golgi structural proteins in various cancer types. Golgi reassembly-stacking proteins (GRASPs) (GRASP65 and GRASP55) and golgin families of proteins contribute to the structural scaffold that defines the Golgi architecture, and are conceived as the Golgi matrix. Altered protein levels of the Golgi matrix are discovered in different cancer types and may facilitate tumor progression. Upward red arrows indicate upregulated protein level, while downward arrows indicate down-regulated protein level of different matrix proteins in different cancer cells. Few studies are correlating the tethering conserved oligomeric Golgi (COG) complex with tumor metastasis, so a question mark is shown.

The multi-compartment stack structure of the Golgi provides an optimal setting for glycosylation. It not only organizes different glycosidases, glycosyltransferases and sugar transporters in an ordered structure and so they can sequentially modify cargo molecules, but also provides each enzyme with an optical microenvironment (e.g., pH, ion, lipid composition, etc.) to maximize its activity (Rivinoja et al., 2012; Wang et al., 2020). Moreover, as the trafficking and processing center of the exocytic and endocytic secretory pathways, the Golgi constantly receives cargo molecules from the ER and endosomes, and thus it needs to maintain its dynamic stack structure to host the Golgi enzymes, and at the same time, allow a constant cargo flow through the Golgi stack at a steady speed. This is not a trivial task. Indeed, there are two seemingly contradictory hypothetical models (and derivatives from each) over the past decades in this field to explain the mechanism of intra-Golgi trafficking, the vesicular transport and the cisternal maturation model. It is possible that both models may not be mutually exclusive and that hybrid models of the two may exist, depending on the cell type and physiological status of the cell. More importantly, both models employ the same set of Golgi building blocks, the Golgi structural proteins, often referred to as the Golgi matrix proteins. Since the first introduction of the concept of “Golgi matrix” (Slusarewicz et al., 1994), several Golgi structural proteins have been identified to be required to maintain Golgi structure and function, including GRASPs and Golgins. Dysregulation of the Golgi structure and Golgi structural proteins has been highly related to glycosylation defects (Hennet and Cabalzar, 2015; Li et al., 2019a). Therefore, it is legitimate to speculate that the dysregulation of Golgi structure may account for the altered glycosylation in tumor progression. The changes of a few critical Golgi structural proteins reported in tumor metastasis are discussed below and depicted in Figure 1.

### GRASP55

Following the establishment of the Golgi matrix hypothesis that Golgi structure formation depends on a network of Golgi structural proteins, Golgi reassembly-stacking protein of 65 kDa (GRASP65) and GRASP55 were identified, characterized, and named for their specific roles in Golgi stack formation (Barr et al., 1997; Shorter et al., 1999). In animal cells, GRASP65 is present in *cis-*Golgi, while GRASP55 is more distributed in the distal Golgi cisternae. Both GRASPs harbor an N-terminal conserved GRASP domain which can form oligomers and glue the adjacent cisternae together into stacks. GRASPs also contain a less conserved C-terminal serine/proline-rich (SPR) domain, which is highly regulated by PTM, such as phosphorylation and *O*-GlcNAcylation (Zhang et al., 2018). Phosphorylation of the C-terminus of GRASPs disrupts the oligomerization property of the N-terminal GRASP domain and thus Golgi stack and ribbon in mitosis, facilitating mitotic Golgi fragment formation (Wang et al., 2003; Wang and Seemann, 2011). Dephosphorylation of GRASPs in telophase recovers the compact Golgi morphology in the peripheral area of the nucleus (Tang and Wang, 2013). Therefore, the featured structures of GRASPs endow them with complementary roles in Golgi stack formation and cisternae-specific ribbon linking with the help of Mena (Tang et al., 2016) and DjA1 (Li et al., 2019b) for GRASP65.

With the stacking role of GRASPs in the Golgi, it is not surprising to see that GRASPs knockdown or knockout reduces the abundance and diversity of global *N*-glycosylation glycan and alters the glycoprotein composition at the cell surface (Xiang et al., 2013; Bekier et al., 2017). The cause of defective glycosylation upon GRASPs depletion can be interpreted in two ways. Firstly, Golgi unstacking enhances vesicle budding, and therefore there is not enough processing time onto the cargos from the glycosylation enzymes (Xiang et al., 2013). Moreover, the cisterna-specific ribbon linking of adjacent stacks is disturbed, and thus the proper Golgi compartmentalization, enzymes localization and glycosylation is violated (Jarvela and Linstedt, 2014). However, it is important to note that there is a discrepancy regarding the role of GRASPs in Golgi cisternal stacking, and recent *in vivo* studies in mouse showed that lack of both GRASPs did not unstack the cisternal core but disconnect the stacks laterally from each other (Grond et al., 2020). The alternative interpretation for this controversy is that the N-terminus of GRASP55 may still be translated in the conditional double knockout (KO) mice and functional for stacking (Zhang and Wang, 2020). Therefore, a proper GRASPs double KO mouse is still needed to fully understand their roles in Golgi architecture. Most recently, acute double depletion of both GRASPs with a degron system also demonstrates that both GRASPs are dispensable for Golgi stacking but mediates Golgi ribbon linking together with GM130 and Golgin-45 (Zhang and Seemann, 2021). Hence, other Golgi stacking factors need further investigation.

In light of the significant roles of GRASPs in Golgi structure formation and proper protein glycosylation, there were speculations between the dysregulation of GRASPs and tumor-associated glycosylation defects. Still, no effect was made to directly correlate GRASPs expression with tumor progression until Kurie and colleagues recently reported that upregulation of GRASP55 was associated with lung adenocarcinoma (LUAD) progression (Tan et al., 2021). Loss of TP53 increased the expression of GRASP55 by silencing miR-34a, a p53 transcriptional target. Upregulated GRASP55 drives tumor progression by enhancing the secretion of pro-tumorigenic effector proteins, such as IGFBP2 and SPP1. Disruption of GRASP55 and Golgin-45 interaction by a small molecule, GRASPIN, inhibits the secretion and reduces the enhanced metastasis caused by TP53-KO in A549 orthotopic lung tumors (Tan et al., 2021). Nevertheless, no glycosylation alterations are investigated in this report which may need further study. It is necessary as GRASPs depletion reduces cell adhesion and accelerates cell proliferation (Ahat et al., 2019), both highly associated with tumor progression. The success of GRASPIN in inhibiting tumor growth suggests a possibility that GRASP55 or its association with crucial proteins may serve as potential therapeutic targets in cancer (Tan et al., 2021).

### GM130

The golgins are a family of predominantly coiled-coil proteins crucial for vesicles tethering to the Golgi (Witkos and Lowe, 2015; Gillingham and Munro, 2016; Muschalik and Munro, 2018). While some golgins are anchored to the Golgi membrane with a signal transmembrane domain at the C-terminus, a majority of them are peripheral membrane proteins that are recruited from the cytoplasm to the cytoplasmic face of the Golgi by association with Rab, Arf, and Arl families small GTPases (Munro, 2011; Kulkarni-Gosavi et al., 2019; Li et al., 2019a). These associations allow the selective localization of a particular golgin to a distinct Golgi sub-compartment (Witkos and Lowe, 2015). Collectively, different golgins localize at specific regions of the Golgi and mediate the tethering of different types of vesicles to specific Golgi subcompartments (Wong and Munro, 2014; Lowe, 2019). For instance, golgins at the *cis-*Golgi mainly tether vesicles coming from the ER, while those at the *trans-*Golgi receive vesicles from the endocytic pathway. Golgins located within the Golgi stack are responsible for intra-Golgi vesicle trafficking. The specificity of vesicle traffic by golgins is one of the mechanisms that the Golgi employs for precise protein processing (Gillingham and Munro, 2016).

GM130 is the first identified Golgi matrix protein and the best-characterized golgin. It is mainly present in the *cis-*Golgi, anchoring adjacent stacks via interaction with GRASP65 at its C-terminus and p115 at its N-terminus (Nakamura et al., 1995; Nakamura et al., 1997; Barr et al., 1998). Depletion of GM130 in culture cells leads to the disconnection of the Golgi ribbon and protein glycosylation defects (Puthenveedu et al., 2006). It has been reported that the GM130–GRASP65 complex directly mediates the localization of the crucial T-synthase (C1GalT1) onto the *cis-*Golgi (Petrosyan et al., 2012). GM130 is attracting considerable interest in the past decade, and accumulating studies support it as a promising anticancer target (Chang et al., 2012). However, there are discrepancies about the relevance between GM130 protein level and tumor progression. Loss of GM130 expression is frequently observed in colorectal and breast cancer patients. Silencing GM130 reduces Cdc42 activity on the Golgi and down-regulates E-cadherin expression, indicating a loss in cell polarity and epithelial identity (Baschieri et al., 2014; Baschieri and Farhan, 2015). GM130 deletion is also associated with increased migration and invasion of breast cancer cells (Baschieri et al., 2015). In contrast, increased invasion and poor prognosis are associated with high levels of GM130 in lung and gastric tumors (Chang et al., 2012; Zhao et al., 2015). Accordingly, downregulation of GM130 decreases angiogenesis and cancer cell invasion, and suppresses tumorigenesis in the lung cancer mice model (Chang et al., 2012). In gastric cancer cells, GM130 depletion increased the level of E-cadherin, which is an epithelial marker but reduced mesenchymal marker, N-cadherin and vimentin, suppressing cell invasion and tumor formation (Zhao et al., 2015). A recent study of the tumor suppressor PTEN’s role in pre-mRNA splicing may support the connection between the high level of GM130 and tumor progression. PTEN deficiency in cancer cells induces aberrant splicing of GM130, resulting in increased GM130 protein level and dramatic Golgi extension and secretion (Shen et al., 2018). Therefore, GM130 levels should be tightly regulated, and alterations in its expression may have adverse effects. This notion is supported by a study that GM130 is a primary target of the Golgi quality control mechanism, the 26S proteasomes mediated Golgi Apparatus-Related Degradation (GARD) (Eisenberg-Lerner et al., 2020).

### Golgin-160 and GMAP210

Golgin-160 and GMAP210 (all called TRIP11) are enriched on the cis-side of the Golgi (Hicks et al., 2006; Cardenas et al., 2009). Golgin-160 has been reported to recruit the dynein microtubule motor to the Golgi, which is crucial for Golgi positioning and structure maintenance (Yadav et al., 2012). By tethering transport vesicles, GMAP210 is engaged in both ER-to-Golgi anterograde and intra-Golgi retrograde trafficking (Roboti et al., 2015). Glial cell line-derived neurotrophic factor (GDNF) triggers glioma cell migration and invasion. Increased expression of golgin-160 and GMAP210 and enlarged Golgi area were observed after GDNF treatment (Tang et al., 2019). Consistently, golgin-160 and GMAP210 depletion reduced the migration and invasion of U251 cells (Tang et al., 2019). In combination, the upregulation of golgin-160 and GMAP210 correlates Gliomas progression, although the detailed mechanisms await further investigation.

### Giantin

Giantin, the largest golgin, is mainly distributed at the rims of Golgi cisternae and inserted into the membrane by a C-terminal transmembrane domain (Linstedt et al., 1995). It functions in laterally linking the Golgi cisternae into ribbon structure (Koreishi et al., 2013). There is decreased level of the extracellular hyaluronan and impaired protein glycosylation in giantin mutant embryos, suggesting its role in proper protein glycosylation (Lan et al., 2016). The Golgi targeting of core 2 glycosyltransferase, C2GnT-L and C2GnT-M, is mediated by giantin (Petrosyan et al., 2012; Petrosyan et al., 2014). Interestingly, defective giantin in prostate cancer cells leads to a shift of glycosyltransferases and α-mannosidase IA from giant in to GM130–GRASP65 site, where the full glycosylation processing is prevented, resulting in high mannose *N*-glycan at the cell surface (Cheng et al., 2020). Therefore, the appearance of cell surface high mannose *N*-glycans may serve as markers of malignant prostate cancer cells (Bhat et al., 2017; Cheng et al., 2020).

### Golgin-97

The *trans-*Golgi network (TGN) is a major sorting station where newly synthesized proteins and lipids are sent out from the Golgi to different destinations. TGN also serves as the entry point for endocytic cargos in retrograde transport. Golgin-97 acts as a scaffold molecule and is recruited onto the TGN by interacting with Arl1. Depletion of golgin-97 impairs the traffic of Shiga toxin subunit B from early endosomes to the TGN (Lu et al., 2004) and blocks the exit of E-cadherin cargo from the TGN (Lock et al., 2005). Low expression of golgin-97 has been reported to correlate with poor patient survival and increased invasiveness in breast cancer. Mechanism investigation showed that Golgin-97 depletion significantly reduces the IκBα protein levels and activates NF-κB, which can promote cell migration and invasion (Hsu et al., 2018).

## Golgi Dispersal as an Indicator of Tumor Progression

The notion that a well-organized Golgi architecture promises its proper function has been widely accepted. Dysregulation of Golgi structural proteins usually correlates with Golgi morphological changes, and Golgi dispersal is frequently observed in various types of cancer cells. Thus, it is simple to speculate that a fragmented or dispersed Golgi morphology may indicate tumor progression. This idea was supported when Golgi phosphoprotein 3 (GOLPH3), the first Golgi resident oncoprotein, was identified (Scott et al., 2009). Compelling evidence demonstrates that tumor cells gain metastatic capacity through a GOLPH3-dependent Golgi membrane dispersal process which enhances vesicular release (Buschman et al., 2015). GOLPH3 is recruited to TGN by binding to phosphatidylinositol-4-phosphate [PI(4)P] in a PITPNC1/RAB1B–containing protein complex dependent manner (Halberg et al., 2016). The PI(4)P-GOLPH3/MYO18A/F-actin module then generates a tensile force that stretches the Golgi membranes and facilitates vesicle budding from the Golgi to the plasma membrane (PM) trafficking (Dippold et al., 2009). Consistently, DNA damage-induced phosphorylation of GOLPH3 by the DNA damage protein kinase (DNA-PK) increases the association between GOLPH3 and MYO18A, leading to enhanced stretching force and thus Golgi dispersal. The altered Golgi morphology and trafficking of cargos to the PM following DNA damage results in cell survival. Therefore, massive Golgi fragmentation has been correlated to tumorigenesis since it is linked to cell survival and resistance to killing by DNA-damaging agents. However, the role of MYO18A/F-actin in Golgi morphology regulation is still controversial, since there is a report that MYO18A does not localize on the Golgi and reduced MYO18A expression does not alter Golgi morphology (Bruun et al., 2017). Hence, further efforts are needed to verify the mechanism of how GOLPH3 modulates Golgi architecture. Other than the morphological effect on the Golgi, recent studies provided evidence that overexpression of GOLPH3 exerts its tumor-promoting activities via enhancing the production of specific growth-inducing glycosphingolipids (GSL). Specifically, GOLPH3 functions as an adaptor between a selectively set of Golgi glycosylation enzymes, especially the GSL biosynthetic pathway enzymes, and COPI coatomer (Eckert et al., 2014; Rizzo et al., 2019). The adaptor role of GOLPH3 mediates the incorporation of GOLPH3 clients into the COPI recycling vesicles, but hinders the clients trafficking to the lysosomes and thus increases the protein levels of glycosylation enzymes (Rizzo et al., 2019). The interaction among GOLPH3 and sialyltransferases also contributes to the oncogenic action of GOLPH3, which efficiently upregulates 2,6-sialylation of β1-integrins and thus enhances integrin-mediated cell migration and signaling pathways (Isaji et al., 2014; Sechi et al., 2020).

However, opposite morphological observations were made in studies on the Golgi during epithelial-to-mesenchymal transition (EMT) of lung cancer cells. Rather than causing Golgi dispersal, EMT led to Golgi compaction with improved ribbon linking and cisternal stacking (Tan et al., 2017). In contrast to GOLPH3, depletion of the Golgi scaffolding protein, PAQR11, disperses the Golgi and impairs anterograde vesicle transport to the plasma membrane. Consistently, the high expression level of PAQR11 is correlated with compact Golgi, EMT, and poor prognosis in human tumors (Tan et al., 2017). An alternative explanation is that EMT is not necessary for metastasis or may be relevant to metastasis in limited tumor types. Collectively, it is still mysterious about the relevance between Golgi morphology and tumor status.

We can envision that the Golgi is a highly dynamic structure, actively sensing and reacting to stress stimuli in the surrounding environment to re-establish Golgi homeostasis for adaption. A systematic approach by RNAi screen demonstrated that depletion of approximately 20% of the signaling genes induced diffused, fragmented, or condensed Golgi. These identified Golgi organization regulators affect the general Golgi functions, including protein secretion and glycan biosynthesis. However, there is not a clear correlation between morphological phenotypes and functional perturbations, indicating a complex network of interaction between signaling cascades and Golgi activities (Chia et al., 2012). The fact is the Golgi structure could be stretched or recovered according to the requirement based on physiological conditions. For instance, the Golgi is extensively fragmented in mitosis and returns to compaction in interphase during the cell cycle (Huang and Wang, 2017). It is also experimentally accurate that the Golgi returns quickly back to normal after drug washout, such as nocodazole (Tang et al., 2016) or Brefeldin A washout (Houghton et al., 2009).

## Outlook

Defective glycosylation of plasma membrane and extracellular matrix has been identified as a hallmark of tumor metastasis for many years. Multiple sets of antibodies have also been developed to detect tumor-associated glycans, while the benefits are still limited due to the tumor heterogeneity and the complexity of glycans (Costa et al., 2020). Therefore, the fundamental reasons for altered glycosylation in cancer cells await deep investigation. As summarized above, even though the exact mechanisms of how GRASP55, Golgin-160, GMAP210, and Golgin-97 regulates protein glycosylation are still missing in cancer cells, GM130, Giantin, and GOLPH3 were characterized to directly modulate specific localization of glycosylation enzymes in the right cisternae, indicating that Golgi structure or Golgi structural proteins probably functions in providing an ordered space for proper protein glycosylation. Thus, it is reasonable to speculate that altered protein levels of Golgi structural proteins might bring about the disorganized distribution of glycosylation enzymes and resultant cancer-related glycosylation defects. However, the elusive role of other unexplored Golgi structural proteins in protein glycosylation regulation needs future investigation. Moreover, the vesicle transportation machinery, such as the retrograde trafficking tethering complex Conserved Oligomeric Golgi (COG), is involved in regulating the correct localization of glycosylation enzymes in Golgi. Multiple mutations in different COG subunits have been identified as a cause for Congenital Disorders of Glycosylation (CDG) in humans (Laufman et al., 2013), but very limited studies reported the correlation between COG expression and cancer progression. In addition, advanced experimental approaches, such as super-resolution microscopy, need to be employed for a precise view of the exact localization of glycosylation enzymes in cancer cells. Therefore, integrative studies by combining glycobiology, cell biology, and clinical investigation are required to generate a complete picture of cancer-related glycosylation and targeted therapy.

The GALA pathway provided evidence that mis-location of Golgi glycosylation enzymes promotes tumor development, nevertheless, the crucial question “does Golgi alteration facilitates oncogenesis” requires deep investigation. Does the Golgi passively respond to the microenvironment of developing tumor and change its morphology and therefore the glycan structure, or actively maintain and recover the original status? To answer this question, more efforts are needed to define the mechanism of Golgi structure formation, function, and response to cellular stresses. Besides, identifying signaling cascades that regulate cell proliferation, membrane trafficking, glycosylation, and tumor progression may result in major breakthroughs in Golgi physiology and tumor pathology.

## Author Contributions

XZ conceived and wrote this manuscript.

## Conflict of Interest

The author declares that the research was conducted in the absence of any commercial or financial relationships that could be construed as a potential conflict of interest.
